# Sphere-Formation Assay: Three-Dimensional *in vitro* Culturing of Prostate Cancer Stem/Progenitor Sphere-Forming Cells

**DOI:** 10.3389/fonc.2018.00347

**Published:** 2018-08-28

**Authors:** Hisham F. Bahmad, Katia Cheaito, Reda M. Chalhoub, Ola Hadadeh, Alissar Monzer, Farah Ballout, Albert El-Hajj, Deborah Mukherji, Yen-Nien Liu, Georges Daoud, Wassim Abou-Kheir

**Affiliations:** ^1^Department of Anatomy, Cell Biology and Physiological Sciences, Faculty of Medicine, American University of Beirut, Beirut, Lebanon; ^2^Department of Biology, Faculty of Arts and Sciences, American University of Beirut, Beirut, Lebanon; ^3^Division of Urology, Department of Surgery, American University of Beirut Medical Center, Beirut, Lebanon; ^4^Division of Hematology/Oncology, Department of Internal Medicine, American University of Beirut Medical Center, Beirut, Lebanon; ^5^Graduate Institute of Cancer Biology and Drug Discovery, College of Medical Science and Technology, Taipei Medical University, Taipei, Taiwan

**Keywords:** sphere-formation assay, prostate cancer, prostatospheres, cancer stem cells, differentiation, self-renewal

## Abstract

Cancer Stem Cells (CSCs) are a sub-population of cells, identified in most tumors, responsible for the initiation, recurrence, metastatic potential, and resistance of different malignancies. In prostate cancer (PCa), CSCs were identified and thought to be responsible for the generation of the lethal subtype, commonly known as Castration-Resistant Prostate Cancer (CRPC). *In vitro* models to investigate the properties of CSCs in PCa are highly required. Sphere-formation assay is an *in vitro* method commonly used to identify CSCs and study their properties. Here, we report the detailed methodology on how to generate and propagate spheres from PCa cell lines and from murine prostate tissue. This model is based on the ability of stem cells to grow in non-adherent serum-free gel matrix. We also describe how to use these spheres in histological and immuno-fluorescent staining assays to assess the differentiation potential of the CSCs. Our results show the sphere-formation Assay (SFA) as a reliable *in vitro* assay to assess the presence and self-renewal ability of CSCs in different PCa models. This platform presents a useful tool to evaluate the effect of conventional or novel agents on the initiation and self-renewing properties of different tumors. The effects can be directly evaluated through assessment of the sphere-forming efficiency (SFE) over five generations or other downstream assays such as immuno-histochemical analysis of the generated spheres.

## Introduction

Stem cells are undifferentiated, self-renewing cells that provide the source of all types of specialized cells in the body, from embryonic development (embryonic stem cells) and throughout adulthood (tissue-specific adult stem cells). These cells are characterized by their self-renewal ability, developmental potency, and their ability to differentiate into different downstream cell lineages. While the zygote, blastomeres, and extraembryonic tissues are totipotent, i.e., able to differentiate into all tissues of the three germ layers, adult stem cells are multipotent cells committed to a specialized lineage. These can include neural stem cells, giving rise to neurons and glial cells; and hematopoietic cells which give rise to different types of blood cells (1). A similar subgroup of self-renewing undifferentiated cells was identified within tumors, showing interesting regenerating ability post-therapy. Based on these findings, the concept of cancer stem cells (CSCs) was developed as a subpopulation of tumorigenic cells, capable of initiating and driving tumorigenesis. The identification of the first CSC in acute myeloid leukemia (AML) in 1994 (2) has given way to potential isolation of similar tissue-specific CSCs and progenitor cells from other tumors (3).

The CSC model proposes a hierarchical organization whereby tumor growth is dependent on CSCs, a presumably small population, that have self-renewal ability and differentiation potential (4–6), thereby giving rise to more differentiated (tumors are mostly anaplastic rather than differentiated) tumor cells, similar to the role of stem cells in normal tissue (7). Another important feature of CSCs is their resistance to cytotoxic chemotherapy and ionizing radiation (8, 9). Some theories have attributed their resistance to their presumably slow cell cycle and overexpression of efflux pumps (10), suggesting that such treatment may instead enrich the CSC sub-population within each tumor (8, 9, 11). Of high clinical relevance becomes the development of novel therapies, specifically targeting CSCs, potentially able to eliminate the regenerating capacity of the tumor.

Cancer stem cells have been identified in many of the solid tumors including brain, breast, prostate, colon, lung, and others. The properties and role of prostate cancer (PCa) stem cells is an active field of research where further investigation is needed to assess the cellular expression of Androgen Receptor (AR) by prostate CSCs, and confirm their ability to give rise to the metastatic form of PCa, namely CRPC (12–19). Therefore, *in vitro* assays that favor the growth and propagation of CSCs is essential to enable their molecular/cellular characterization. Lately, it has been shown that CSCs have the ability to form multicellular three-dimensional (3D) spheres *in vitro* when grown in non-adherent serum-free conditions (20, 21). Such 3D cultures allow the growth and propagation of CSCs, as well as evaluating the potential use of various conventional and novel drugs to target these tumor-initiating cells (21, 22). However, most of the currently used protocols for 3D culturing of tumor spheroids in suspension exhibit forced floating and hanging drop approaches for screening of drugs (21, 23–25), which display several limitations and challenges pertaining efficient assessment of the number and size of cultured spheres, as they are mobile and can merge with one another (24, 26).

The time, cost, and technical challenge of performing self-renewal *in vivo* studies highlight the need to develop alternative methods. Hence, *in vitro* sphere-forming assays have been established to investigate PCa (27, 28), similar to those developed to study the nervous system (29) and mammary glands (30). Spheres with self-renewing properties formed in a 3D culture matrix which resembles the native microenvironment can be generated from human and mouse prostate epithelial cells. The sphere formation assay (SFA) provides a useful tool to assess the stem cells' population residing in tumors or cancerous cell lines and screen for drugs specifically targeting CSCs.

Here, we report the methodology for generating and propagating prostate spheres (prostatospheres) from murine prostate tissue and from human and murine PCa cell lines. This method has been previously used to generate prostate spheres from primary murine PCa cells (31–36), as well as human and murine-derived PCa cell lines (32, 35, 37). The protocol described herein proposes a semisolid Matrigel™-based 3D culturing system which overcomes previously mentioned limitations of 3D culturing of spheroids in suspension and hence prevents the migration and fusion of spheres. Besides, a new regimen of treatment is designated in the context of CSCs where spheres can be treated over several generations, consecutively or alternatively, to better evaluate and screen for drugs and anti-cancer agents. This *in vitro* 3D culturing technique provides a functional reporter of stem/progenitor cell activity in PCa cell lines.

## Materials and equipment

### Reagents

- RPMI-1640 AQmedia™ (Sigma, cat. no. R2405)
- DMEM AQmedia™ (with 450 mg glucose/L) (Sigma, cat. no. D0819)
- Prostate epithelial cell growth media, PrEGM BulletKit (Lonza, cat. no. CC-3165 & CC-4177)
- Keratinocyte growth media, KGM Bullet Kit (Lonza, cat. no. CC-3101 & CC-4131)
- Fetal Bovine Serum (FBS; Sigma, cat. no. F9665)
- MEM Non-Essential Amino Acid Solution (100X) (NEAA; Sigma, cat. no. M7145)
- Sodium Pyruvate 100 mM Solution (Sigma, cat. no. S8636)
- Penicillin-Streptomycin (Lonza, cat. no. DE17-602E)
- Plasmocin™ Prophylactic (InvivoGen, cat.no. ant-mpp)
- Dulbecco's PBS without Calcium and Magnesium (D-PBS; Sigma, cat. no. D8537)
- Collagenase Type II (Worthington; cat. no. LS004174)
- Gelatine powder (Fluka, cat. no. 48723)
- 0.05% Trypsin/EDTA (Sigma, cat. no. T3924)
- Dispase (Life Technologies, cat. no. 17105-041)
- Trypan Blue Solution (0.4%) (Sigma, cat. no. T8154)
- Growth factor-reduced Matrigel™ (BD Biosciences, cat. no. 354230)
- Isoflurane (Abbott, cat. no. B506)
- 0.5% Triton X-100 (Biorad, cat. no. 1610407)
- Bovine Serum Albumin (BSA; Amresco, cat. no. 0332)
- Normal Goat Serum (NGS; Invitrogen, cat. no.16210064)
- Fluoro-gel II with DAPI (Electron Microscopy Sciences, cat. no. 17985-50)
- Permount mounting media (Invitrogen, cat. no.P36934)
- TRIZOL Reagent (Ambion, cat. no. 15596018)
- Y-27632 dihydrochloride (Santa Cruz, cat. no. sc-281642)
- UltraPure™ DEPC-Treated Water (ThermoFisher SCIENTIFIC, cat. no. 10813012)
- Mouse monoclonal anti-CD44 (Santa-Cruz, cat. no. sc-7297)
- Mouse monoclonal anti-SOX2 (Santa-Cruz, cat. no. sc-365823)
- Mouse monoclonal anti-CD117 (BD Pharmingen, cat. no. 555713)
- Rat monoclonal anti-CD49f (BD pharmingen, cat. no. 555734)
- Alexa 488 goat anti-mouse (ThermoFisher SCIENTIFIC, cat. no. A-11001)
- Alexa 633 goat anti-rat (ThermoFisher SCIENTIFIC, cat. no. A21094)
- Ethanol (Sigma, cat. no. 793213)
- Paraformaldehyde (Sigma, cat. no. 441244)
- 3% Hydrogen peroxide (Sigma, cat. no. H6520)
- Sodium azide (Sigma, cat. no. 199931)
- RIPA buffer (Sigma, cat. no. R0278)
- PolyFreeze Tissue Freezing Medium (Sigma, cat. no. SHH0026)
- Citric Acid (Sigma, cat. no. 251275)
- DMEM Harvest media (see Reagent Setup)
- RPMI Harvest media (see Reagent Setup)
- PrEGM media (see Reagent Setup)
- KGM media (see Reagent Setup)
- DMEM Dissection media (see Reagent Setup)
- RPMI Dissection media (see Reagent Setup)
- KGM dissection media (see Reagent Setup)

### Equipment

- 15 mL conical tubes (Corning, cat. no. 430791)
- 50 mL conical tubes (Corning, cat. no. 430829)
- Syringes and needles (BD Biosciences)
- 40 μm pore size nylon mesh filter (BD Biosciences)
- Microtubes (1.5 mL; Thermo Fisher Scient, cat. no. AB0620)
- Tissue culture dish (100 mm; Corning, cat. no. 430167)
- Tissue culture plate (24 well; Corning, cat. no. 3526)
- Tissue culture plate (12 well; Corning, cat. no. 3513)
- Tissue culture plate (6 well; Corning, cat. no. 3516)
- Cryovials (1.5 mL; Corning, cat. no. 431386)
- Bright-Line™ Hemocytometer (Sigma, cat. no. Z359629)
- CoolCell™ LX Freezing Container (Sigma, cat. no. BCS-405)
- Cell culture incubator set at 37°C, 5% CO_2_ (ThermoFisher SCIENTIFIC)
- Centrifuge (ThermoFisher SCIENTIFIC)
- Shaking water bath
- Spinning wheel
- Inverted microscope
- Confocal microscope (Zeiss LSM 710)
- Viva View FL Incubator Microscope (Olympus Life Science)

### Reagents setup

- **DMEM Harvest medium:** To prepare 100 mL of DMEM harvest medium, mix 87 mL of the basal DMEM AQmedium™ or RPMI-1640 AQmedia™ with 10 mL of FBS, 1 mL of penicillin-streptomycin, 1 mL of NEAA (100X) and 1 mL of sodium pyruvate (100 mM). Filter the medium with a 0.22-μm filter unit and store it for up to 4 weeks at 4°C.
- **RPMI Harvest medium:** To prepare 100 mL of RPMI harvest medium, mix 87 mL of the basal RPMI-1640 AQmedium™ with 10 mL of FBS, 1 mL of penicillin-streptomycin, 1 mL of NEAA (100X) and 1 mL of sodium pyruvate (100 mM). Filter the medium with a 0.22-μm filter unit and store it for up to 4 weeks at 4°C.
- **PrEGM medium:** To prepare 100 mL of PrEGM medium, mix 87 mL of the basal PrEGM medium™ with 10 mL of FBS, 1 mL of penicillin-streptomycin, 1 mL of NEAA (100X) and 1 mL of sodium pyruvate (100 mM). Filter the medium with a 0.22-μm filter unit and store it for up to 4 weeks at 4°C.
- **KGM medium:** To prepare 100 mL of KGM medium, mix 87 mL of the KGM medium with 10 mL of FBS, 1 mL of penicillin-streptomycin, 1 mL of NEAA (100X) and 1 mL of sodium pyruvate (100 mM). Filter the medium with a 0.22-μm filter unit and store it for up to 4 weeks at 4°C.
- **DMEM Dissection medium:** To prepare 100 mL of dissection medium, mix 100 mL DMEM Harvest media with 1 mg/mL collagenase I. Store it for up to 4 weeks at 4°C.
- **RPMI Dissection medium:** To prepare 100 mL of dissection medium, mix 100 mL RPMI Harvest media with 1 mg/mL collagenase I. Store it for up to 4 weeks at 4°C.
- **KGM dissection medium:** To prepare 100 mL of KGM dissection medium, mix 100 mL of KGM media with 0.5 mg/mL dispase and 0.5 mg/mL Collagenase I. Store it for up to 4 weeks at 4°C.

**Note: Artifact:** Propagating prostatospheres for several generations over 2 months in culture may predispose cultured cells to mycoplasma contamination. Plasmocin™ Prophylactic can be added to the media; its use has no significant effects on sphere culture conditions. The use of Plasmocin™ can be omitted if the user plans on culturing the prostatospheres for one generation only.

### Stepwise procedures

This study was carried out in accordance with the recommendations of the NIH Guide and the American University of Beirut Guidelines for Use and Care of Animals. The protocol was approved by the Institutional Animal Care and Utilization Committee of the American University of Beirut.

For sphere-formation assay, prostate cells can be prepared from murine prostate tissues and from human and murine PCa cell lines.

**Figure 1:**
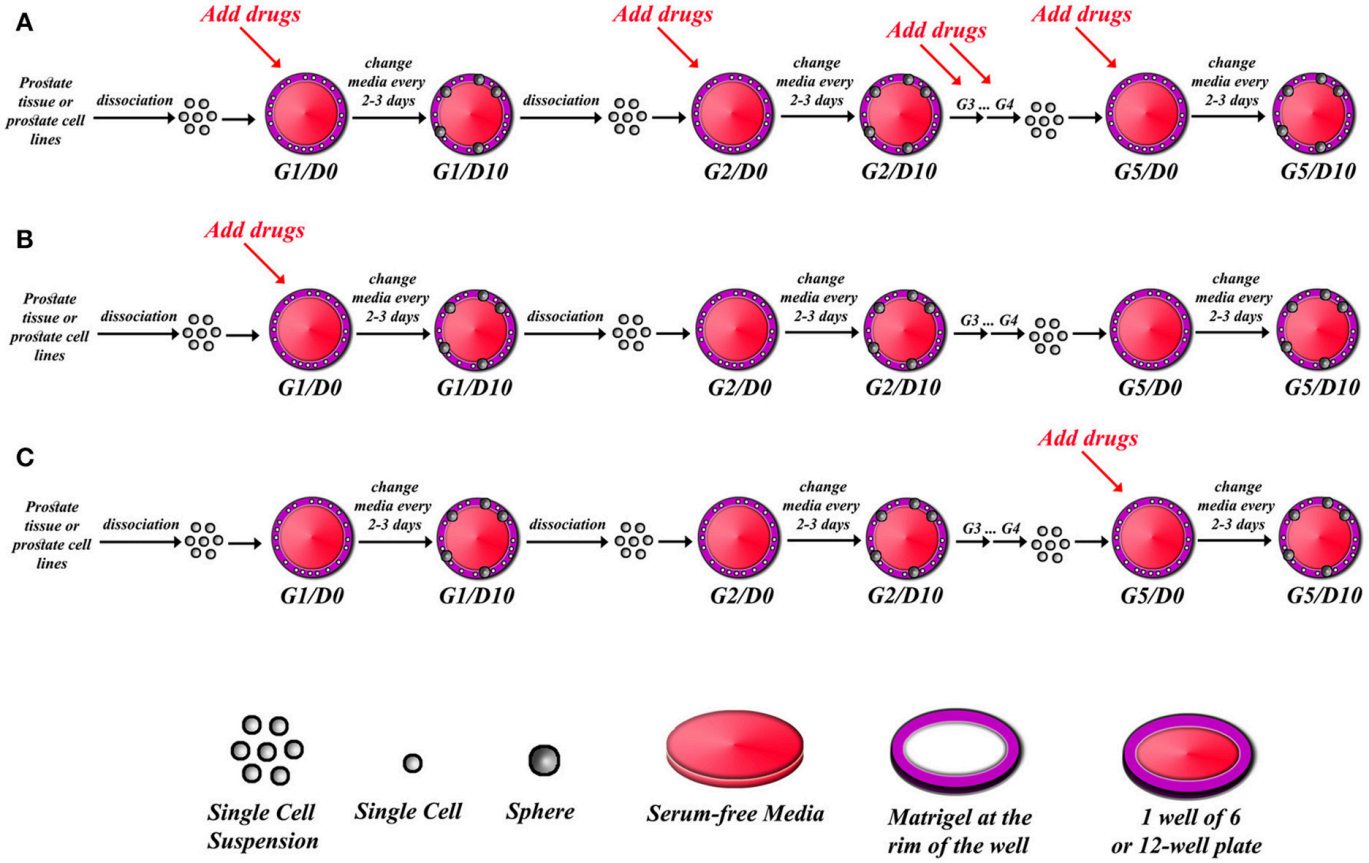
Schematic illustrating strategy of drug treatments in sphere-formation assay. After isolating single cell suspension from PCa tissues or PCa cell lines, drugs can be added to every generation in the sphere-formation assay **(A)**, or drugs can be added to the first generation of spheres only (G1D0) and then spheres can be serially propagated to investigate whether the effect is permanent or reversal **(B)**, or spheres can be formed and serially passaged so that you have extensively grew and enriched for stem cells, and then drugs are added at G5D0 to potentially target those cells **(C)**. The sphere-formation efficiency has to be calculated for each generation to assess self-renewal ability of sphere-forming cells. At each generation, spheres could be processed for immune-histochemical analysis to check for differentiation markers, or proteins and total RNA could also be extracted to assess for differentiation.

**Figure 2:**
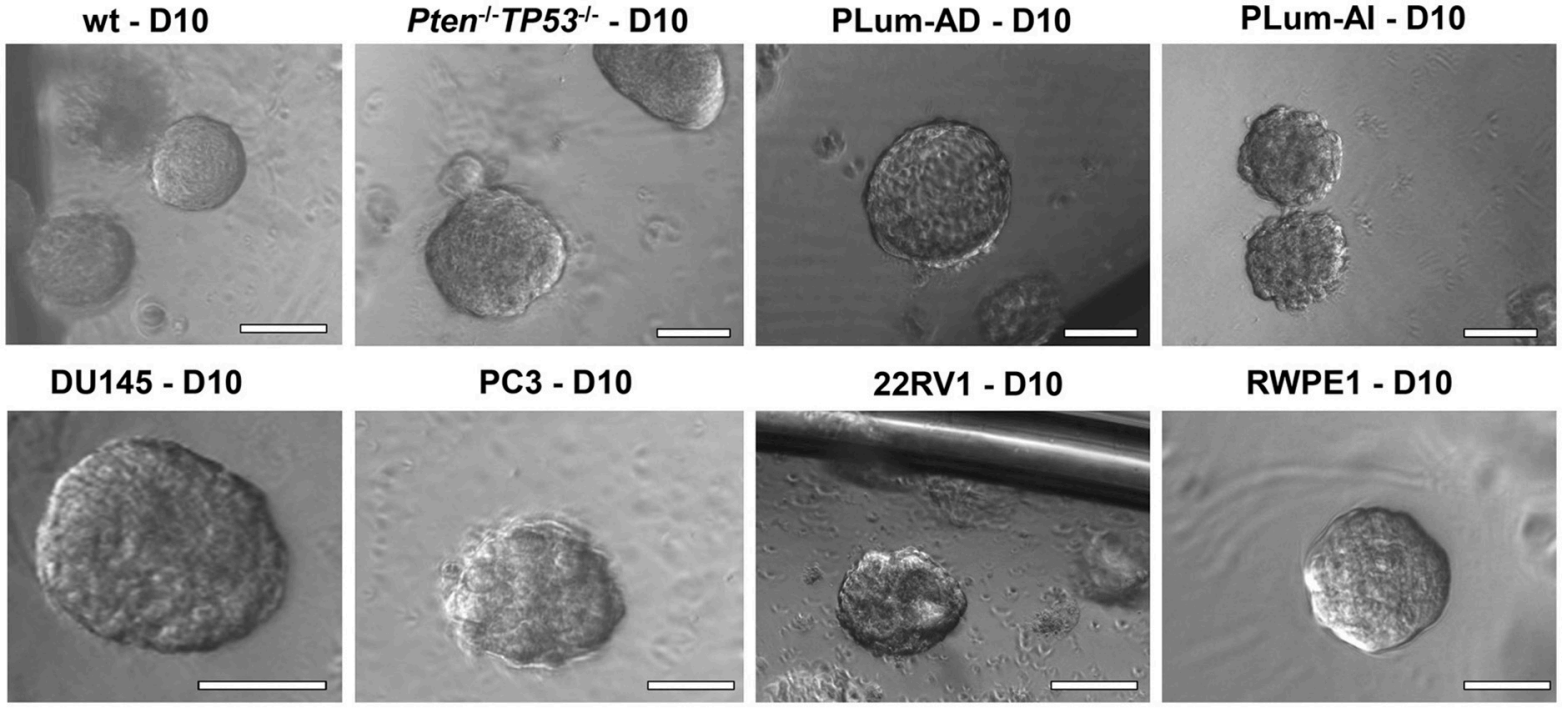
The sphere-forming assay with murine and human prostate cells. Representative bright-field images of mouse *wt, Pten*^−/−^*TP53*^−/−^, PLum-AD, and PLum-AI prostate spheres (**upper panel**) and human DU145, PC3, 22RV1, and RWPE1 (**lower panel**) prostate spheres are shown. Zeiss Axiovert microscope was used for the acquisition of bright field images. Scale bars = 100 μm.

**Figure 3:**
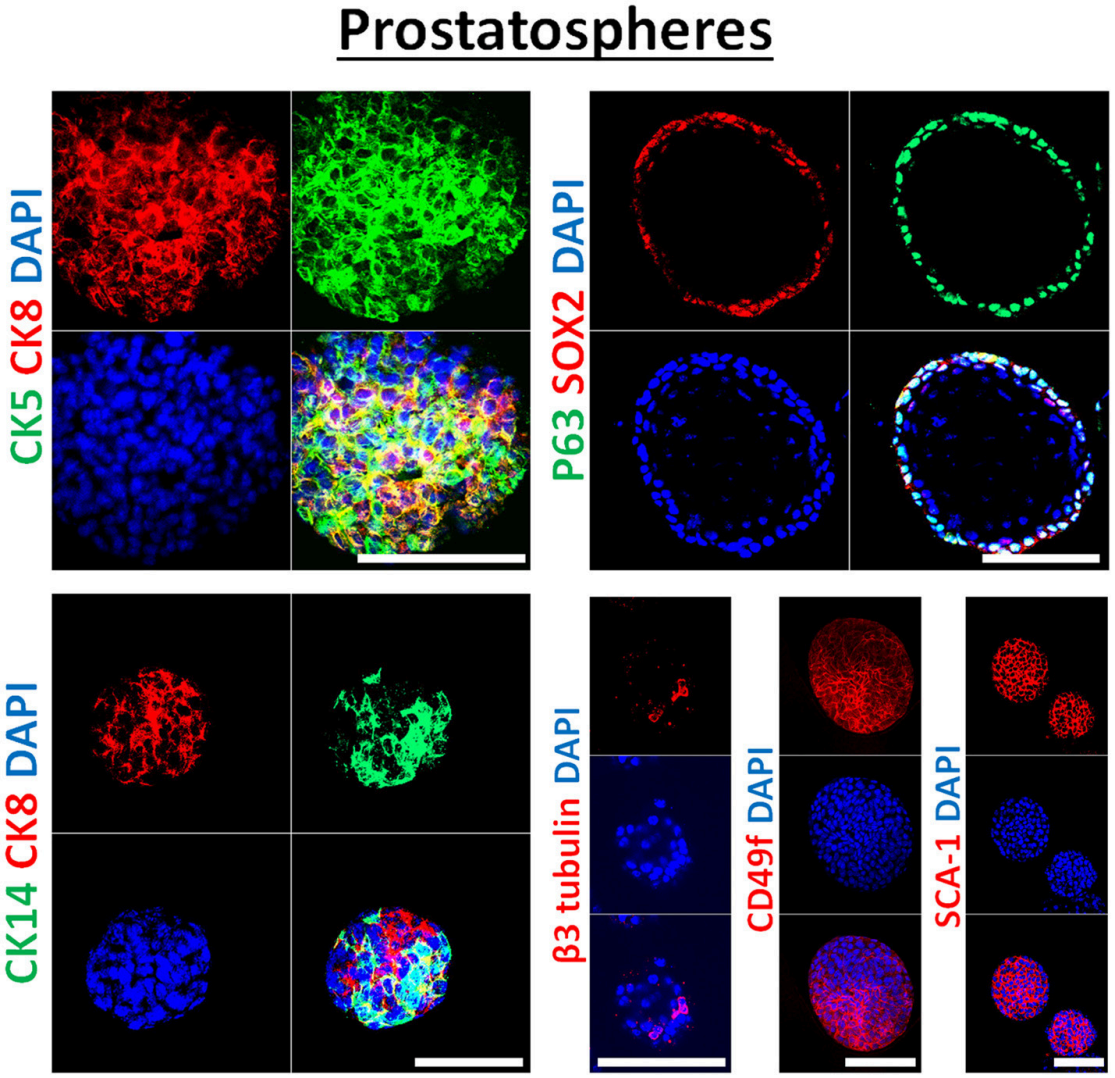
Immunophenotype of prostatospheres. Immunofluorescent images of confocal cross sections from mouse *wt* prostate spheres stained for prostate lineage epithelial markers CK8, CK5, and CK14 (left panel) and stem cell markers: p63, SOX2, CD49f, and SCA-1 (right panel). Prostatospheres displayed a heterogenous population of cells displaying intermediate cytokeratin profiles, where major population of cells co-expressed CK8 (luminal prostate cell marker) and either CK5 or CK14 (basal prostate cell marker). Furthermore, positive expression of the neuroendocrine marker β3 tubulin was detected in a minor population of cells within the prostatospheres. On the other hand, co-expression of p63 [basal prostate cell marker and believed to be a marker of the stem cells of developing prostate epithelium (46)] and SOX2 [essential embryonic stem cell gene involved in prostate tumorigenesis (47)] was also detected, besides expression of the stem cell marker CD49f and SCA-1, which have been shown to identify putative prostate stem-like cells (48, 49). The nuclei were stained with anti-fade reagent Fluorogel II with DAPI. Scale bars = 100 μm. Representative confocal microscopy images were acquired using the 63x oil objective and images were processed using the Zeiss ZEN 2012 image-analysis software. Microscopic analysis was performed using Zeiss LSM 710 laser scanning confocal microscope (Zeiss). DAPI, 40,6-diamidino-2-phenylindole.

1. **Isolation of primary prostate cells from murine prostate tissues (Timing** ~ **2 h)**
  1.1. Sacrifice the male mouse (8-12 weeks old) using isoflurane inhalation followed by cervical dislocation.
    1.1.1. Spray the mouse with 70% ethanol to minimize contamination.
    1.1.1. Lay the mouse on its dorsal side and make a horizontal incision, followed by a vertical one just above the urethra.
    1.1.3. Remove the urogenital system (UGS) by pulling up on the bladder and cutting the connective tissue below it. This enables you to obtain the bladder, seminal vesicles, prostate, urethra, and part of ureters. Place the dissected parts of the UGS in a 100-mm Petri dish with Dulbecco's Modified Eagle's Medium (DMEM) Harvest media.
      **Note: Caution:** Wear gloves when dissecting and change gloves after isolating UGS parts or if the gloves become contaminated.
  1.2. Using a dissecting light microscope, isolate the different lobes of the mouse prostate.
    1.2.1. Remove all the fat chunks (soapy appearance) attached to the UGS, then gently peel off the anterior prostate lobes from the seminal vesicles and cut off the seminal vesicles and discard them.
    1.2.2. Cut off what remains of the ureters with a fine scissor.
    1.2.3. Hold on to the urethra using sterile tweezers and start breaking apart the dorsolateral and ventral lobes of the prostate. At this stage, the prostatic lobes can be treated as separate entities to isolate cells separately from anterior lobes, ventral lobes, and dorsolateral lobes, or transferred altogether to a new 60-mm petri dish supplied with DMEM dissection media.
      **Note: Potential pitfall:** All following steps should be carried out in a primary cell culture laboratory under a cell culture hood (i.e., Class II biosafety cabinet) using sterile reagents and equipment while wearing gloves.
      **Note:** For further details on the dissection and extraction of the adult murine prostate, please refer to the manuscript published by Oliveira et al. discussing the anatomy of the procedure on mouse models (38).
  1.3. Mince the tissue with a sterile scalpel blade into very small pieces of around 0.3–0.5 mm in size and then transfer the minced tissue into a 15 mL conical tube. Conveniently, use 3 mL of DMEM dissection media for each dissected prostate gland. Treat tissues with Collagenase Type II (5 mg/mL, freshly prepared), with ROCK pathway inhibitor Y-27632 (10 μM in DMEM dissection media). Incubate the mixture in a shaking water bath or spinning wheel at 37°C for a minimum of 1 h.
    **Note:** The shaking shall be gentle to avoid any physical damage to the cells.
  1.4. Spin down the minced tissue at 200 × g for 5 min. Aspirate supernatant and add 2 mL of pre-warmed 0.05% Trypsin-EDTA solution. Add ROCK pathway inhibitor Y-27632 (10 μM). Resuspend the pellet and place it in a humidified incubator at 37°C for 5 min.
  1.5. Neutralize the trypsin by adding 2 mL of DMEM harvest media containing 5% of Fetal Bovine Serum (FBS) and then spin down at 200 x g for 5 min at room temperature.
  1.6. Aspirate the supernatant, then resuspend the pellet in 3 mL of prostate epithelial cell growth media (PrEGM).
  1.7. Start the mechanical dissociation by passing the mixture gently through a series of needles with different gauges. We usually start by 19-gauge, then 21-gauge, then 23-gauge, then 25-gauge.
  **Note: Artifact:** At this stage we take a 10 μL sample of the mixture and place it on a glass slide to check the status of the cells under a light microscope. Check for the presence of any clumps of cells or tissue in the mixture as those might generate artifacts.
  **Note: Troubleshooting:** If there are still cell clumps, we continue by passing the cells through a 27-gauge needle, followed by a 30-gauge needle. Sometimes, in case of big tumors or big chunks, the 23 or 25-gauge needle becomes clogged. In this case, we pass the mixture through a nylon mesh filter of a 40 μm pore size into a 50 mL conical tube and then transfer the mixture to a 15 mL conical tube and continue as mentioned above.
  1.8. Make sure cells are properly dissociated and of appropriate morphology. Spin down the cells at 200 x g for 5 min, aspirate the media, wash the cells once with 3 mL of PrEGM. Resuspend the pellet in 1 mL of PrEGM and count the cells using a hemocytometer and trypan blue (to differentiate living cells from dead ones).
  **Note:** the previous steps discussing the isolation of primary prostate cells from murine prostate tissue shall be done as fast as possible, to preserve survival of these cells.
2. **Isolation of cells from prostate cancer cell lines (Timing** ~ **30 min)**
  2.1. Aspirate the growth media from a 100 mm dish of adherent monolayer prostate cells. Wash once with warm D-PBS.
  2.2. Add 1 mL of pre-warmed 0.05% Trypsin-EDTA solution. Incubate in a humidified incubator at 37 °C for at least 3 min.
  2.3. Observe the cells under the microscope for detachment. If cells are less than 90% detached, increase the incubation time a few more minutes, checking for dissociation every 30 s. You may also tap the flask to expedite cell detachment. When ≥ 90% cells are detached, neutralize the trypsin with 1 mL of the cell line's appropriate growth media containing 5–10% FBS.
  2.4. Transfer into a 15 mL conical tube and spin down the cells at 200 x g for 5 min. Resuspend the cells in 3 mL of growth media to start the mechanical dissociation.
    **Note: Artifact:** avoid any clumps. At this stage, take a 10 μL aliquot of the mixture and place it on a glass slide to check the status of the cells under a light microscope. Starting from a cell line, more than 90% single cell suspension is usually obtained after trypsinization. If clumps are present, pass the cell suspension through a 25-gauge needle gently and check again. At this stage, you should have a clear single cell suspension. Spin down the cells at 200 x g for 5 min, aspirate the media, resuspend in 1 mL of serum-free growth media. Count the cells using a hemocytometer and trypan blue.
3. **Seeding cells for sphere-formation assay (Timing** ~ **1.5 h)**
  3.1. After counting the single cell suspension of the freshly isolated cells from tissues or cell lines (see above), cells can be kept on ice until ready to be used.
    **Note:** Cells can be kept on ice for up to 1 h before use.
  3.2. Regular or growth factor-reduced Matrigel™ is thawed on ice before you start the cell isolation procedure.
    **Note: Troubleshooting:** A bottle of Matrigel™ shall be thawed on ice, 4 to 5 h before performing the experiment. Aliquots of 1 ml shall be prepared to prevent repeated freeze/thaw cycles of Matrigel™.
  3.3. To generate spheres from primary prostate cells, plate 2,000-10,000 cells/well in a 12-well plate. To generate spheres from PCa cell lines, plate 1,000-5,000 cells/well in a 12-well plate.
    **Note: Troubleshooting:** the number of cells seeded per well can be optimized for each prostate cell line through seeding a serial dilution of cells across different wells. The optimal number of cells to be seeded should be balanced between a countable number of generated spheres and a large amount of seeded cells to validate the results. a total of 100 μL mix (50 μL cold cell suspension: 50 μL cold Matrigel™) for each well of a 12-well plate. Use 1:1 ratio of cell suspension in Serum-free media to Matrigel™.
    **Note: Troubleshooting:** Double the amount is needed for every well of a 6-well plate (i.e., 200 μL mix), and half the amount is needed for a 24-well plate (i.e., 50 μL mix). If you are plating in duplicate or triplicate, always prepare a master mix according to the formula of n+1. (For example, to plate three wells of a 12-well plate, prepare a master mix of 300 + 100 = 400 μL (200 μL cell suspension: 200 μL Matrigel™).
    **Note: Troubleshooting:** Always keep the mix of cells and Matrigel™ on ice in order to prevent the solidification of Matrigel™. Make sure to pipet up and down to keep the cells uniformly suspended before plating. Perform gentle pipetting to avoid forming any bubbles in the Matrigel™ during the process.
    **Note: Artifact:** Matrigel™ Matrix/Cell suspension (1:1 mixture) is used as a semisolid serum-free matrix to avoid adherence of the cells and promote sphere formation from single cells. Once formed, spheres are less likely to migrate and aggregate, which is in contrast to spheres growing in suspension. Other semisolid matrices may be used, depending on the requirements of the used cell lines, including collagen-based matrices (39) or hydrogel-based matrices (40).
  3.5. Slowly pipet 100 μL around the bottom rim of a well in a 12-well plate uniformly by going around in a circular manner.
    **Note: Troubleshooting:** Avoid forming bubbles as bubbles will create gaps in the Matrigel™ that will lead to its breakage. To get rid of a bubble, carefully prick it with the needle of a 27-gauge syringe. A uniform and bubble-free plating can be confirmed under light microscopy. Three pivotal reasons root for plating the matrix around the rim of the well. First, it allows feeding and washing from the center of the empty well without disturbing the Matrigel™ matrix on the rim. Second, the rim provides edge support for the Matrigel™ mix thereby ensuring physical stability for the period of sphere formation that might last for 2 weeks. This is important since Matrigel™ is a gel-like substance that tends to become very loose and to break whenever plated in the center of the well (especially that spheres are left in culture for up to 2 weeks). Therefore, this is significantly avoided by plating the Matrigel™ matrix around the rim. Third, it confines the spheres' growth to a defined area in the well that makes the task of counting spheres much easier and more precise. In addition, it insures the distribution of cells in a single-cell format, avoiding clumping and overgrowth.
    **Note: Potential pitfall:** As a possible alternative, cells could be plated in the middle of the well (and not all over the well to minimize the use of Matrigel™, which is another reason why plating around the rim is preferred). Although cells might be crowded in that way, but still possible to do. In this case, and if opted to plate in the middle, semi-automated counting could be achieved via acquisition of images along the z-axis and in a tile scan manner to cover the whole area and the different optical sections i.e., thickness of Matrigel™.
  3.6. Allow the Matrigel™ to solidify in a humidified incubator at 37°C for 45–60 min.
    **Note: Troubleshooting:** Avoid tilting the plate while plating and transferring the plate to the incubator. After solidification, slowly add 1 mL of warm serum-free media (PrEGM, RPMI, or KGM) or low-serum (1% FBS) media, with or without a specific drug, in the middle of each well. It is very important to use warm media as cold media will lead to Matrigel™ dissociation.
  3.7. Replenish with warm media as in the original plating (with or without drug) every 2–3 days. It is very important to aspirate the media from the center of the well very gently as to not to disturb the Matrigel™.
    **Note: Troubleshooting:** Matrigel™ cell suspensions must be followed up daily to check up over spheres growth and formation. In general, spheres will start to appear by day three or four. Each cell line exhibits a distinct pattern in the process of spheres formation and growth, which must be monitored to optimize the follow-up schedule. It is very important to periodically acquire bright field images, using an inverted light microscope, of developing spheres to assess for any changes in morphology.
    **Note:** if the user is interested in assessing the effects of certain drugs on the sphere forming ability, a proper amount of the drug must be used in specific wells and compared to control wells with the appropriate media. We and others have shown the effects of many drugs, targeting specific signaling pathways, on sphere formation ability of presumably putative cancer stem/progenitor cells (31, 32, 34, 36, 41–44). There are many potential strategies of assessing the effect of a drug or a novel therapeutic agent in an SFA setting as outlined in Figure 1. A continuous treatment over five generations can indicate whether the effect of a drug is cumulative or not. Alternatively, we can establish and propagate spheres over five generations without treatments to enrich for cancer stem/ progenitor cells, followed by treatments at G5 to assess if a drug can target this enriched cell population or not.
  3.8. Spheres can be counted starting day 8–10 and the sphere formation efficiency (SFE) or sphere formation unit (SFU) can be calculated based on this simple formula: **SFE** = (number of spheres counted ÷ number of seeded cells) × 100. For example, the formation of 20 spheres at day 10 after plating 2,000 cells indicates an SFE of 1%. In addition, bright-field images showing morphology of mature spheres can be taken and documented starting day 8–10 as illustrated in Figure 2.
    **Note: Pause Point:** Note that the spheres that are formed in the primary sphere-formation assay, referred to as generation 1 spheres (G1), do not all account for stem cells, as progenitor cells can form short-lived spheres too. Because of that, multiple serial propagations of the spheres in the Matrigel™ are essential to truly identify stem cell characteristics (self-renewal ability). Thus, after counting the number of spheres and calculating the SFE, spheres propagation shall follow.
    **Note: Troubleshooting:** since the formed spheres are embedded in a 3D Matrigel™ matrix, an automated method to count spheres using the microscope has been proven hard to develop due to the different optical sections of every sphere. Nonetheless, the geometrical distribution of spheres around the rim of the well significantly facilitates the manual counting. This allows for defining a starting point using a marker at the bottom of the well and go in a full circle minimizing the error of counting the same sphere twice. It is hence much harder when Matrigel™ is plated in the center where each well will have to be split into quadrants to be able to count.
4. **Propagating prostatospheres (Timing** ~ **8 weeks)**
  4.1. Aspirate the medium from the center of the well and add to each well 500 μL of dispase solution dissolved in the spheres' appropriate growth media (at a final concentration of 1 mg/mL). Incubate for a minimum of 30 min in a humidified incubator at 37°C. Dispase will break down the Matrigel™ and will release the spheres into the media.
  4.2. Collect the media with the released spheres and centrifuge at 200 x g for 5 min.
  4.3. Resuspend the pellet in 1 mL of 0.05% Trypsin-EDTA and incubate in a humidified incubator at 37°C for 10 min.
  4.4. Neutralize trypsin by adding 1 mL of growth media containing 5% FBS, and transfer into 15 mL conical tube.
  4.5. Centrifuge the cells at 200 x g for 5 min, aspirate the supernatant, and then resuspend the pellet in 1 mL of prostate growth media. Start the mechanical dissociation process by passing them through a series of needles with different gauges following the exact same procedure as explained before (1.7).
    **Note: Artifact:** In the case of any doublet in G1 sphere formation (which should be very minimal if any), this will not impact the propagation of spheres as propagation involves the complete dissociation of spheres into single cells.
  4.6. Centrifuge cells at 200 x g for 5 min, resuspend in fresh sphere growth media and count the cells using a hemocytometer and trypan blue.
    **Note: Potential pitfall:** Propagation shall be repeated to a minimum of 5 generations of spheres (G5). This is a very important step as to verifying the extensive self-renewal ability of stem cells (45).
    **Note: Troubleshooting:** the SFE of each cell line has to be calculated through manual counting of the spheres, as stated in section 3.8. The number of sphere-forming units (SFUs) is dynamic and might change from one generation to another and from one cell type to another throughout the 5 generations starting from the same amount of cells at the start point (22, 31, 32, 34–37). This is used to assess the self-renewal ability of the CSCs.
    Each sphere presumably originated from a single stem/progenitor cell and therefore, the differentiation potential within spheres should be tested. To do so, spheres could be collected at any generation (preferably at each generation), and could be subjected to immunohistochemistry and immunofluorescence analysis as explained before (31, 32) and as shown in Figure 3. These methods can be used to further assess the effects of used treatments on differentiation pathways of targeted tumors/cells. Furthermore, total protein and RNA can be extracted from treated and non-treated spheres at any generation and subjected to WB and qRT-PCR analyses of the stemness and differentiation markers (41).
5. **Immunofluorescence staining (Timing** ~ **2 days)**
  5.1. Spheres are grown in 35-mm glass bottom culture plates with 10-mm microwell in Matrigel™ -containing media following the same protocol as described above.
  5.2. Using a pasteur pipette, aspirate the media from the center not to disturb the Matrigel™.
  5.3. Rinse with 1 mL of sterile D-PBS, and then gently aspirate using the same procedure.
  5.4. Fix the spheres by adding 0.5 mL of 4% paraformaldehyde (PFA) for 20-30 min at room temperature.
  5.5. At the end of the incubation, aspirate the PFA solution.
  5.6. Wash with 1 mL of D-PBS, and leave for 3 min. This washing procedure is repeated three times to remove any excess PFA.
    **Note: Pause Point: Fixed Spheres can be kept at 4**°**C up to 1 month before proceeding with the following steps**.
  5.7. After aspirating the D-PBS, permeabilize the fixed spheres with 0.5% Triton X-100 in D-PBS for 30 min at room temperature.
  5.8. Wash three times with 1 mL of sterile D-PBS, for 3 min each.
  5.9. After carefully aspirating the permeabilization solution, block the spheres by using a blocking buffer (0.1% Bovine Serum Albumin (BSA), 0.2% Triton X-100, and 10% Normal Goat Serum (NGS) in D-PBS) for 2 h at room temperature.
  5.10. Wash three times with 1 mL of sterile D-PBS, for 3 min each.
  5.11. Aspirate the D-PBS. Incubate the spheres with 200 μL of the sphere blocking buffer containing the primary antibody for 1 h or leave it overnight at room temperature.
  5.12. Remove the primary antibody after incubation time. Gently wash the spheres three times with 1 mL of D-PBS containing 0.1% Tween-20.
  5.13. Incubate the spheres with 200 μL of the sphere blocking buffer containing the secondary antibody (Alexa-488 and/or 568 conjugated IgG) for 2 h at room temperature.
    **Note: Potential pitfall:** From this point on, the experiment must proceed in the dark. Keep the slides wrapped in aluminum foil paper.
  5.14. Aspirate the secondary antibody and wash gently with 1 mL of D-PBS containing 0.1% Tween-20. This washing procedure is repeated three times at room temperature.
  5.15. Gently wipe excess D-PBS on the slide then add 10 μL of mounting medium composed of freshly prepared anti-fade reagent Fluoro-gel II with DAPI.
  5.16. Mount gently using a 12-mm glass coverslip (avoid making bobbles).
  5.17. Seal the edges of each cover slip by spreading a small volume of nail polish to avoid air-drying.
  5.18. Examine under confocal laser scanning microscope or epifluorescent microscope.
6. **Staining of frozen sections (Timing** ~ **30 min)**
  6.1. After pelleting the intact spheres (see step 2 in the propagating prostatospheres assay), wash the spheres with 1 mL of D-PBS and spin down at 200 g for 5 min at room temperature.
  6.2. Directly embed the spheres pellet in PolyFreeze Tissue Freezing Medium (stored at −80°C).
  6.3. Fasten the embedded block to the block holder in the cryostat at −20°C.
  6.4. Section the frozen spheres block into desired thickness (typically 5 to 10 microns).
  6.5. Mount the sphere sections on chilled, pre-cleaned, uncoated microscope glass slides (Sections can be stored in a sealed slide box at −80°C for later use).
  6.6. Apply staining methods as described above.
7. **Spheres protein extraction (Timing** ~ **1 h)**
  **Note:** Precool the centrifuge to 4°C.
  7.1. After pelleting the intact spheres (see step 2 in propagating prostatospheres assay), wash the spheres with 1 mL of D-PBS and spin down at 200 x g for 5 min at room temperature.
  7.2. Aspirate the D-PBS then resuspend the pellet in 150 μL of freshly prepared 1x lysis buffer (RIPA buffer).
  7.3. Incubate on ice for 15 min.
  7.4. Centrifuge at 2,400 x g for 20 min at 4°C.
  7.5. Transfer the supernatant to a new tube.
  7.6. Boil the samples for 10 min at 95°C.
  7.7. Store at −20°C for later use.
8. **Spheres RNA extraction using TRIZOL (Timing** ~ **1 h)**
9. **Note:** Precool the centrifuge to 4°C.
  8.1. After pelleting the intact spheres (see step 2 in the propagating prostatospheres assay), wash the spheres with 1 mL of D-PBS and spin down at 200 x g for 5 min at room temperature.
  8.2. Lyse the spheres by adding 1 mL of TRIZOL reagent by repetitive pipetting and passing through a syringe 27- or 28-gauge needle.
  8.3. Transfer the cell suspension into a 1.5 or 2 mL eppendorf.
  8.4. Incubate the samples for 5 min at room temperature.
  8.5. Centrifuge the spheres for 1 min at 2,600 x g at 4°C (to remove cell debris).
  8.6. Collect the supernatant and add 200 μL of chloroform.
  8.7. Vortex the samples vigorously for 15 s then incubate at room temperature for 2-3 min.
  8.8. Centrifuge the samples at 12,000 x g for 15 min at 4°C.
  8.9. Transfer the upper aqueous phase carefully without disturbing the inter-phase into a new eppendorf.
  8.10. Add 0.5 mL of isopropanol alcohol to the aqueous phase and incubate at room temperature for 10 min.
  8.11. Centrifuge at 12,000 g for 10 min at 4°C, and then remove the supernatant completely.
  8.12. Wash twice with 1 mL 75% ethanol by resuspending and pelleting at no more than 7,500 g at 4°C.
  8.13. Air dry the tubes by leaving them on the bench for the ethanol to evaporate for 5-10 min
  8.14. Dissolve the RNA pellet in 100 μL diethyl pyrocarbonate (DEPC)-treated water.
  8.15. Store the samples at −80°C for later use.
    **Note: Troubleshooting:** depending on the used cell lines, the generation number following one/several propagations, the culturing conditions, and the used treatment, the number and the size of the spheres may vary, affecting the yield of extracted RNA. The use of RNeasy Kit by Qiagen is recommended over Trizol extraction.

### Timing

1. Isolation of primary prostate cells from murine prostate tissues (Timing ~ 2 h)
2. **Note: Troubleshooting:** isolating primary prostate cells from murine models shall be done as fast as possible to promote survival of these cells.
3. Isolation of cells from PCa cell lines (Timing ~ 30 min)
4. Seeding cells for sphere-formation assay (Timing ~ 1.5 h) prostatospheres (Timing ~ 8 weeks)
5. Immunofluorescence staining (Timing ~ 2 days)
6. Staining of frozen sections (Timing ~ 30 min)
7. Spheres protein extraction (Timing ~ 1 h)
8. Spheres RNA extraction using TRIZOL (Timing ~ 1 h)

## Anticipated results

In this manuscript, we successfully generated prostate spheres from different human PCa cell lines (22RV1, RWPE1, PC3, and DU145). Using the above protocol, we also generated prostate spheres from primary single cell suspensions obtained from *wt* and *Pten*^−/−^*TP53*^−/−^ mouse prostate tissues as demonstrated in Abou-Kheir et al. (31, 32), Agarwal et al. (36), and Daoud et al. (37), and from our previously established murine PCa cells, which represent androgen-dependent PCa and CRPC, namely PLum-AD and PLum-AI, respectively, as in Daoud et al. (37). In addition, *TMPRSS2-* driven *ERG* expression has been shown to increase the self-renewal and expand the numbers of clonogenic self-renewing CRPC subpopulation progenitors, as assayed by *in vitro* prostatospheres formation assay in Casey et al. (34). Representative images of the spheres generated from different cell lines, as well as primary murine prostate epithelial cells are presented in Figure 2.

Single cell suspensions of prostate cells either from mouse (Figure 2, upper panel) or human cell lines (Figure 2, bottom panel) were mixed with Matrigel™ (1:1) and then plated at a density of 5,000 cells/well in a 24-well plate. Media was changed every 2–3 days and bright field images were taken at days 8–12. Spheres were successfully propagated for at least 5 generations to assess the self-renewal ability of the stem/progenitor cells population. Single cells were monitored for single sphere formation over several days. The use of Matrigel™ semisolid matrix (or other semisolid matrices, as suggested in the procedure) allows seeded cells to remain embedded in place and keeps them less likely to migrate and move within the matrix. This is practically useful to overcome several limitations seen in prostate spheroids cultured in suspension, which tend to coalesce, merge, and therefore require very low plating density (as low as 1 cell per well), to form clonal colonies. Louis et al. (39) mirrored this issue in neurospheres formed in suspension as compared to others formed in semisolid collagen-based matrix, stating similar advantages to the applied 3D-culture (39). Hereby, we present a high-resolution live imaging movie of individual *wt* murine PCa cells embedded in a semisolid Matrigel^TM^-based 3D culturing system, showing their behavior in forming spheres, each of which is derived from a single cell (Video S1).

The number of spheres were counted and compared to the initial number of seeded cells. The percentage of sphere-forming units (SFU) within each cell line, i.e., the ratio of average number of prostatospheres to the initial number of seeded cells, is representative of the stem/progenitor cells subpopulation in culture. The average SFU seen using primary cells taken from murine prostate is 0.5%, while the average SFU using prostate cell lines is 5–10%, as seen in El-Merahbi et al. (35). The average SFU of each cell line is consistent over the 5 generations, which marks the ability of these cells to self-renew.

In our previous work on *wt* and *Pten*^−/−^*TP53*^−/−^ primary prostate cells (32) and murine PCa cell lines PLum (37), generated spheres were characterized through immunofluorescent staining, showing the expression of lineage markers of basal (CK5), luminal (CK8) and neuroendocrine cells (β3 tubulin) of prostate tissue. In this manuscript, the generated spheres from human PCa cell lines were stained for epithelial cell markers (CK5, CK8, CK14, and β3 tubulin), as well as stem cell markers (p63, SOX2, CD49f, and SCA-1). Figure 3 shows selective immunofluorescence images of stained prostatospheres derived from primary *wt* murine prostate cells. Interestingly, the spheres generated from those cells have self-renewal ability when subjected to a minimum of five propagations, and after staining of the prostatospheres at G0 and G5, no significance difference was detected in the expression levels (31, 32, 36, 37).

Due to the heterogenic character of PCa cell lines, and the lack of universal markers for CSC in prostate tumors, generated spheres from all human and murine PCa cell lines were stained for an array of CSC markers (50) including: CD44, SOX2, CD117 (c-kit), and CD49f (Figure S1). The levels of expression of different CSC markers (CD44, CD133, SSEA4, c-kit, NKx3.1, OCT-4, CD49f, and CD24) was further assessed by measuring their respective mRNA levels using qRT-PCR, on the spheres derived from human and murine PCa cell lines. As expected, each cell line presented a selective combination of stem cell markers: for example, PC3-derived spheres showed relatively high expression of CD44, CD133, and SSEA4 (compared to non-significant expression of other markers), while DU45-derived spheres showed an increased expression of c-kit (CD117), NKx3.1, and OCT4 (Figure S2, Table S1).

This assay can further be used as a functional reporter of the progenitor activity of different cell lines, as well as the differentiation and self-renewal ability of the stem/progenitor cell population (27). This property provides a platform to test the effects of traditional and novel therapeutic agents on PCa cell lines. We have previously used this assay to compare the effects of selective inhibitors of the AKT/mTOR pathway (Rapamycin and Triciribine), as well as the downstream signaling of the androgen receptor (Nilutamide and bicalutamide) on wild-type and *Pten*^−/−^*TP53*^−/−^ mouse prostate spheres (31). The effect of these drugs was monitored through measuring the sphere-forming efficiency (SFE) and the average volume of the formed spheres; which were both decreased compared to non-treated control prostatospheres. Similarly, we studied the effects of Ehrenb extracts on the sphere-formation ability of PCa cell lines DU45 and PC3. The decreased ability to form spheres on G1, and the limited self-renewal ability during propagation of these spheres over 5 generations as outlined in Figure 1, correlated with the ability of this extract to limit cellular growth, migration, and proliferation on 2D-functional assays (35).

## Discussion

In this manuscript, we outline a protocol to generate and propagate prostate spheres (prostatospheres) from prostate tissues and human and murine PCa cell lines. This assay depends on the ability of stem cells to survive in a 3D culture matrix (for example, Matrigel™) in low or serum-free medium while differentiated cells grow in adherent monolayer cultures and FBS-containing medium. Primary plating of single-cells suspension in a 3D matrix doesn't confirm the existence of an enriched stem cell sub-population, which is characterized by its self-renewal ability and differentiation potential. Multiple and serial propagations of the spheres are needed to confirm the existence of CSCs within a tumor or a cell line, dependent on finding a relatively constant SFU/SFE within each generation.

The issue of self-renewal represents a central feature of CSCs and is tightly associated with their pathologic ability to regenerate tumors after treatment. It represents the ability of these cells to reproduce indefinitely, while maintaining its multipotent ability to differentiate. 45 discussed this issue in terms of propagation and self-renewal of neuronal adult stem cells, cultured as neurospheres in suspension. (45) They properly argue that non-stem progenitor cells retain the ability of self-renewal and can indeed propagate into secondary or tertiary spheres (G2 and G3, respectively). Theoretically, prostate stem cells shall maintain self-renewal ability indefinitely. Due to technical limitations faced *in vitro*, propagating prostate cell-derived spheres over five generations (or more) can be practical to isolate and propagate prostate CSCs; the sphere-forming ability of progenitor cells decreases subsequently, while that of prostate CSCs is maintained.

The clonal origin of the formed spheres is yet another important aspect of this assay. Previous work showed the tendency of neurospheres to coalesce and merge when cultured in suspension. In order to generate clonal colonies using cells grown in suspension to culture neurospheres, deposition of a single cell per well presented the golden criteria to reach that goal (39). Previous studies have successfully cultured prostatospheres in serum-free suspension culture, starting from PCa cell lines (51, 52). However, this issue hasn't been properly addressed. The protocol proposed herein suggests growing of cells in semi-solid matrix, to avoid their migration and aggregation, and overcome the technical burden of culturing non-adherent spheres starting from single cell per well. Discrete spheres were grown and monitored starting from a single cell suspension (Video S1). Matrigel™ provides an enriched matrix that harbors different glycoproteins and growth factors seen in the basement membrane, including collagen IV, laminin, and Fibroblast Growth Factor; it provides a semi-solid matrix able of simulating the rich extracellular medium of cells *in vivo* (53). The use of Matrigel™ has been further implicated in long term organoids culture, another 3D-culturing modality that closely mirrors the *in vivo* prostate of murine and human derived cells (54). Moreover, Matrigel™ has been used to facilitate the establishment of tumorigenic Xenografts derived from human cell lines in immunocompromised mice (53).

A previous study found that the 3D solid spheres of mammary glands, cultured in Matrigel™, could be used for repopulating gland-free mammary fat pads in mice reaching an engraftment frequency of 71% (55). This means that the spheres formed can be used for tissue transplants and that this assay could be further developed in the future to be applicable on human tissue grafts. Interestingly, it was also found that the sphere assay could be used to compare the cellular mechanisms of normal and malignant cells through deciphering active pathways and differentiation patterns. It was found that the spheres derived from normal lung tissue, cultured in Matrigel™, were able to form a lumen while the spheres derived from cell lines and tumor biopsies of lung cancer were not (56), showing a potential use of the sphere-formation assay to compare the underlying cellular processes governing CSCs and those in normal tissue stem cells, and their pathophysiological implications on progression of the disease. Another study, by Hur et al. found that hematospheres could be isolated from blood and cultured in Matrigel™ to allow the formation of a network of vessels (57), proposing potential therapeutic implications to treat coronary artery occlusions or atherosclerotic arteries, by vessel replacement therapies.

Because the use of SFA has been recently implemented on assessing stem cell-like properties in tumors, there has not been much focus on drug treatments targeting these self-renewing cells. However, there have been various studies that focused on testing particular drugs on generated spheres. SFA was used, along with different assays, to show that Nigericin, an antibiotic that suppresses Golgi function in eukaryotic cells, suppresses colorectal cancer metastasis by inhibiting epithelial-mesenchymal transition (EMT) (58). The drug Metformin was shown to inhibit the growth of thyroid carcinoma cells, suppress the self-renewing property of the thyroid CSCs and act as an enhancing supplement to chemotherapeutic agents (43). Furthermore, Eckol has been proven to suppress the maintenance of stem cell properties and malignancies in glioma stem-like cells, hence suggesting a reduced chance of cancer recurrence (42). Also, spheres from esophageal tumor origin have been found to involve stem-like cells with elevated aldehyde dehydrogenase activity (59). *Berberis libanotica Ehrenb* (BLE) extracts as well were shown to have high therapeutic potential in targeting PCa and eradicating the self-renewal ability of highly resistant CSCs (35). All the mentioned examples have used SFA to assess the effect of drugs on the CSCs or stem cell-like populations in tumors; these studies primarily relied on two main criteria in their assessment: average volume of the generated spheres and the average number of sphere-forming units (SFU). Yet, most studies focus on testing particular drugs on generated spheres for single generation, without assessing the effect on spheres propagated for several generations, which possess enriched stem cell/progenitor properties.

To emphasize the importance of the use of SFA in stem cell and cancer research, we have put together a detailed protocol of how to conduct a semisolid Matrigel™-based sphere-formation assay in the absence or presence of drugs, consecutively or alternatively, in the context of PCa stem cells. The advantage of using this sphere-formation *in vitro* assay is to isolate, propagate, purify, and amplify specific populations of prostatic normal and CSCs. It enables studying stem cells at different stages of their formation (at different generations) and detecting markers of their signaling pathways. It also solely depends on the functional intrinsic property of stem cells in forming a complex structure in a 3D environment, namely self-renewal ability and differentiation potential.

## Author contributions

HB, KC, RC, OH, AM, and FB contributed to the project design and execution of experiments, analysis of results, and writing of manuscript. AE-H, DM, Y-NL, GD, and WA-K contributed to overlooking and following up with experiments, result analysis and manuscript proofreading. Y-NL, GD, and WA-K contributed to project design, result analysis, manuscript writing and proofreading. All authors critically revised and edited the manuscript and approved the final draft.

### Conflict of interest statement

The authors declare that the research was conducted in the absence of any commercial or financial relationships that could be construed as a potential conflict of interest.

## Supplementary material

Figure S1 Immunophenotyping of spheres derived from human and murine prostate cancer cell lines. Immunofluorescent images of PC3, DU145, 22RV1, RWPE-1, PLum-AI, PLum-AD derived prostatospheres, stained for selected cancer stem cell markers including CD44, SOX2, CD117, and CD49f (white arrows). Those cancer stem cell markers have been shown to identify putative prostate stem-like cells (47, 49, 60, 61). CD49f stain (white arrows) is displayed in the middle of some prostatospheres in addition to the cells that express this marker at the surface. Noteworthy, in some cases, CD49f is ubiquitously expressed at the surface of the spheres (as in RWPE-1, DU145, and PC3), which is due to the presence of basal epithelial cells that are mostly found at the outer layers of the sphere. CD44, on the other hand, is a widely used cell surface marker that is expressed in many cells including stem cells (62) and was found to be homogeneously expressed across the different prostatosphere cells (as shown in the images to the left). The nuclei were stained with anti-fade reagent Fluorogel II with DAPI. Representative confocal microscopy images were acquired using the 63x oil objective and images were processed using the Zeiss ZEN 2012 image-analysis software. Microscopic analysis was performed using Zeiss LSM 710 laser scanning confocal microscope (Zeiss). Scale bar = 20 μm.

Figure S2 Relative mRNA expression of cancer stem cell markers in prostatospheres. The relative mRNA expression of CD44, CD133, SSEA4, c-Kit, NKx3.1, and OCT-4 in prostatospheres derived from human-derived prostate cancer cell lines (PC3, DU45, RWPE1, and 22RV1), and the relative mRNA expression of CD49f and CD24 in prostatospheres derived from murine prostate cell lines (PLum-AI and PLum-AD), as assessed by qRT-PCR. GAPDH expression was used as a reference gene. (Please refer to Table S1 for primers sequence).

Table S1 List of Human and Mouse primers for real-time PCR.

Video S1 Recorded growth of prostatospheres in Matrigel™ matrix. This high-resolution movie shows the growth of single cell suspensions of *wt* murine prostate into individual spheres. This technique overcomes the limitations of culturing prostate spheroids in suspension, by limiting the migration and aggregation of generated spheres. This time-lapse movie of prostate sphere-formation assay was captured hourly over 100 h using an Olympus Viva View FL Incubator Microscope with a 40x objective (Olympus).

## Abbreviations

CSCs: cancer stem cells
PCa: prostate cancer
CRPC: castration-resistant prostate cancer
SFA: sphere-formation assay
SFE: sphere-forming efficiency
AML: acute myeloid leukemia
AR: androgen receptor
3D: three-dimensional
IACUC: Institutional Animal Care and Use Committee
UGS: urogenital system
DMEM: Dulbecco's Modified Eagle's Medium
FBS: fetal bovine serum
PrEGM: prostate epithelial cell growth media
PFA: paraformaldehyde
BSA: bovine serum albumin
NGS: normal goat serum
DEPC: diethyl pyrocarbonate
CK5: cytokeratin 5
CK8: cytokeratin 8
CK14: cytokeratin 14
E-cad: E-cadherin
EMT: epithelial-mesenchymal transition
BLE: berberis libanotica ehrenb.
