# Supplementary figures and images for: Sphere-Formation Assay: Three-Dimensional in vitro Culturing of Prostate Cancer Stem/Progenitor Sphere-Forming Cells
Source: Front Oncol. 2018 Aug 28;8:347. doi: 10.3389/fonc.2018.00347 (PMC6121836; doi:10.3389/fonc.2018.00347)

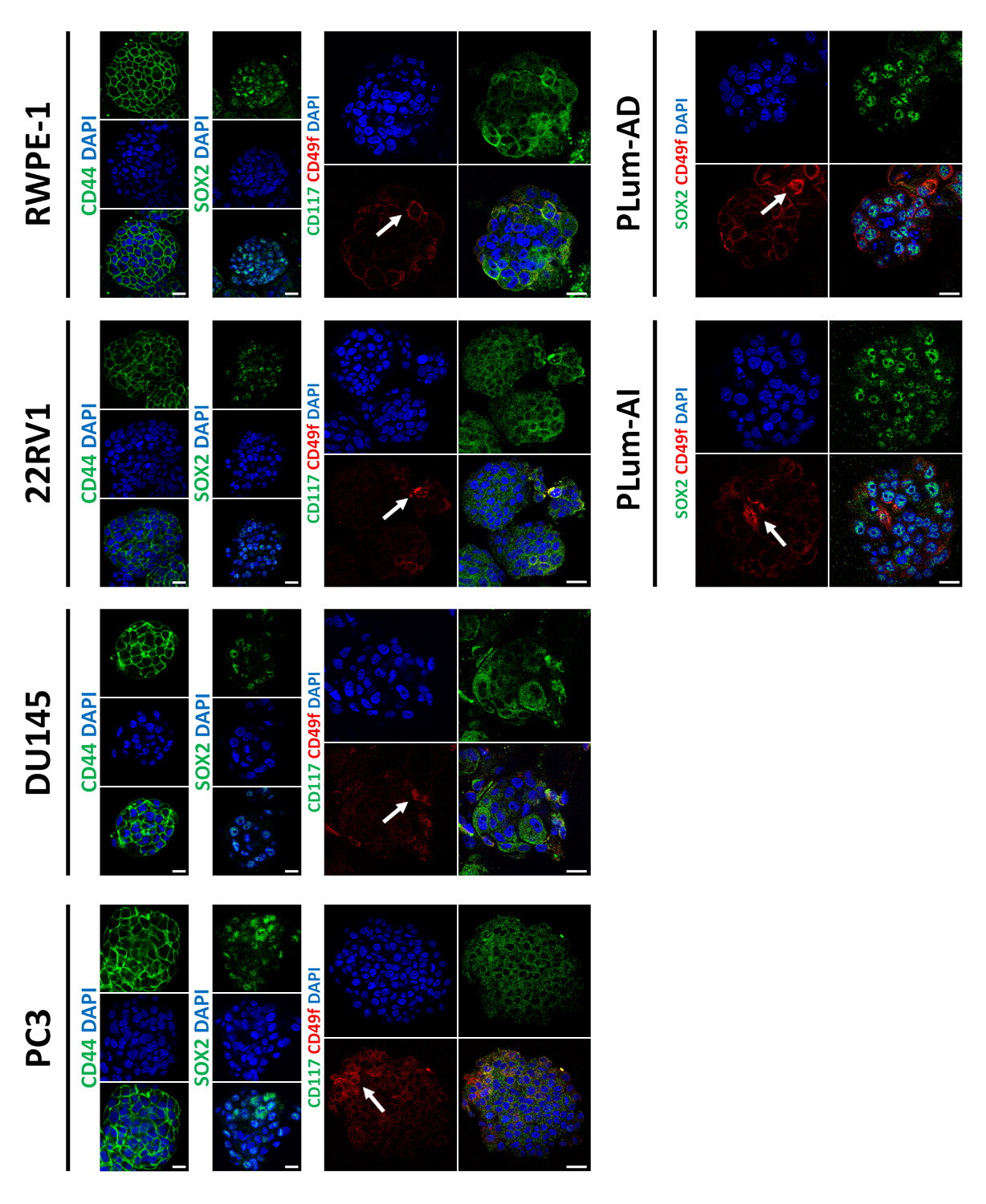

Supplement: Figure S1 — Immunophenotyping of spheres derived from human and murine prostate cancer cell lines. Immunofluorescent images of PC3, DU145, 22RV1, RWPE-1, PLum-AI, PLum-AD derived prostatospheres, stained for selected cancer stem cell markers including CD44, SOX2, CD117, and CD49f (white arrows). Those cancer stem cell markers have been shown to identify putative prostate stem-like cells (47, 49, 60, 61). CD49f stain (white arrows) is displayed in the middle of some prostatospheres in addition to the cells that express this marker at the surface. Noteworthy, in some cases, CD49f is ubiquitously expressed at the surface of the spheres (as in RWPE-1, DU145, and PC3), which is due to the presence of basal epithelial cells that are mostly found at the outer layers of the sphere. CD44, on the other hand, is a widely used cell surface marker that is expressed in many cells including stem cells (62) and was found to be homogeneously expressed across the different prostatosphere cells (as shown in the images to the left). The nuclei were stained with anti-fade reagent Fluorogel II with DAPI. Representative confocal microscopy images were acquired using the 63x oil objective and images were processed using the Zeiss ZEN 2012 image-analysis software. Microscopic analysis was performed using Zeiss LSM 710 laser scanning confocal microscope (Zeiss). Scale bar = 20 μm. [file Image_1.TIF]

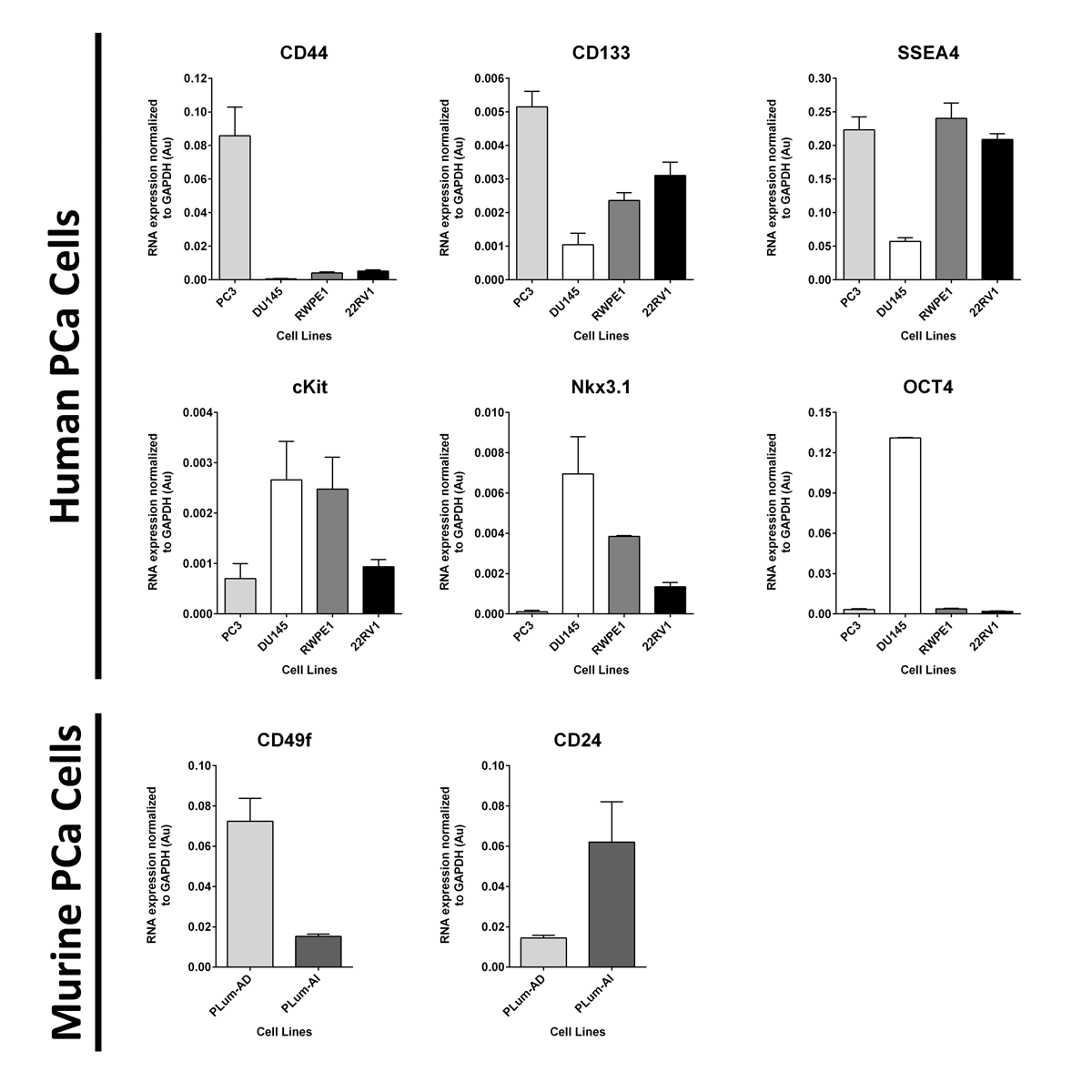

Supplement: Figure S2 — Relative mRNA expression of cancer stem cell markers in prostatospheres. The relative mRNA expression of CD44, CD133, SSEA4, c-Kit, NKx3.1, and OCT-4 in prostatospheres derived from human-derived prostate cancer cell lines (PC3, DU45, RWPE1, and 22RV1), and the relative mRNA expression of CD49f and CD24 in prostatospheres derived from murine prostate cell lines (PLum-AI and PLum-AD), as assessed by qRT-PCR. GAPDH expression was used as a reference gene. (Please refer to Table S1 for primers sequence). [file Image_2.TIF]
