# Supplementary material for: Sphere-Formation Assay: Three-Dimensional in vitro Culturing of Prostate Cancer Stem/Progenitor Sphere-Forming Cells
Source: Front Oncol. 2018 Aug 28;8:347. doi: 10.3389/fonc.2018.00347 (PMC6121836; doi:10.3389/fonc.2018.00347)
Supplement: Table S1 — List of Human and Mouse primers for real-time PCR. [file Table_1.DOCX]

| **Primer** | **Sequence** |
| --- | --- |
| Human CD44-F | 5’TTTGCATTGCAGTCAACAGTC3’ |
| Human CD44-R | 5’GTTACACCCCAATCTTCATGTCCAC3’ |
| Human CD133-F | 5’GATTAAGTCCATGGCAACAGCG3’ |
| Human CD133-R | 5’GCTGGTCAGACTGCTGCTAAGC3’ |
| Human Oct4-F | 5’AGAACATGTGTAAGCTGCGG3’ |
| Human Oct4-R | 5’GTTGCCTCTCACTCGGTTC3’ |
| Human SSEA4-F | 5’GAGCTCACAGCAGGATAGGC3’ |
| Human SSEA4-F | 5’GGGCACTGTGTCCAATACCA3’ |
| Human c-Kit-F | 5’CAGGCAACGTTGACTATCAGT3’ |
| Human c-Kit-R | 5’ATTCTCAGACTTGGGATAATC3’ |
| Human NKx3.1-F | 5’CAGAGACCGAGCCAGAAAGG3’ |
| Human NKx3.1-R | 5’CTGAGTGTGGGAGAAGGCAG3’ |
| Mouse CD49f-F | F: 5’AGAGACATGAAGTCCGCGCA3’ |
| Mouse CD49f-R | R: 5’ACCTTCCCCAGATCATCATAG3’ |
| Mouse CD24-R | 5’ACACACTGATGCTTACCCGT |
| Mouse CD24-F | 5’GTAGCTTCGGGTCTGTGAGC |

**Table S1. List of Human and Mouse primers for real-time PCR.**
